# A Dual-input deep learning architecture for classification and latency estimation in ABR signals

**DOI:** 10.3389/fmed.2025.1693921

**Published:** 2025-11-11

**Authors:** Youssef Darahem, Oguz Yilmaz, Halil B. Saldirim, Berna Mutlu, Hasan F. Ates, Bahadir K. Gunturk

**Affiliations:** 1 Department of Computer Engineering, Istanbul Medipol University, Istanbul, Türkiye; 2 Department of Audiology, Bakircay University, Izmir, Türkiye; 3 Department of Health Sciences, Istanbul Medipol University, Istanbul, Türkiye; 4 Department of Artificial Intelligence and Data Engineering, Ozyegin University, Istanbul, Türkiye

**Keywords:** auditory brainstem response (ABR), deep learning, convolutional neural networks (CNNs), transfer learning, machine learning (ML)

## Abstract

**Introduction:**

Auditory brainstem response (ABR) is an objective neurophysiological evaluation designed to measure the electrical activity originating from the auditory nerve and brainstem in response to auditory stimulation. This assessment objectively records synchronous neural activity as it propagates along the auditory pathway. It is characterized by several distinct waves, most notably waves I, III, and V. Wave V plays a central clinical role since its presence and latency are routinely used to assess a patient's hearing status. However, manual identification and localization of wave V are time consuming and subjective. Previous work has explored automated detection methods to reduce this burden.

**Methods:**

In this paper, we make two primary contributions. First, we propose a multi-task deep learning pipeline that simultaneously (i) detects the presence of wave V and (ii) predicts its latency, thus eliminating the need for separate manual interpretation steps and enhancing clinical usability. Second, inspired by the audiologist's practice of comparing responses at multiple click sound intensities—specifically, using responses at high intensities, where waves are more prominent, as reference—we introduce a paired-signal approach. Each input to our deep learning model consists of the test signal together with its corresponding 80 dB reference from the same recording session. This provides the model with richer contextual information, and we show that the paired-signal approach improves over the single input approach. For multi-task learning, we design a network that consists of a backbone and two branches, one for latency prediction and the other for classification of whether wave V exists or not. We first train a latency-prediction network and then freeze its feature extraction layers to initialize a classification branch. Finally, we fine-tune the entire network using a joint loss function that balances classification and regression objectives.

**Results:**

Experimental results demonstrate that our joint model^1^ outperforms conventional single-task approaches. For classification, it achieves an F1-score of 0.92; for latency regression, it attains an *R*^2^ of 0.90.

**Discussion:**

Our findings highlight the promise of convolutional neural networks for enhancing ABR analysis and underscore their potential to streamline clinical workflows in the diagnosis of auditory disorders.

## 1 Introduction

**Figure 1:**
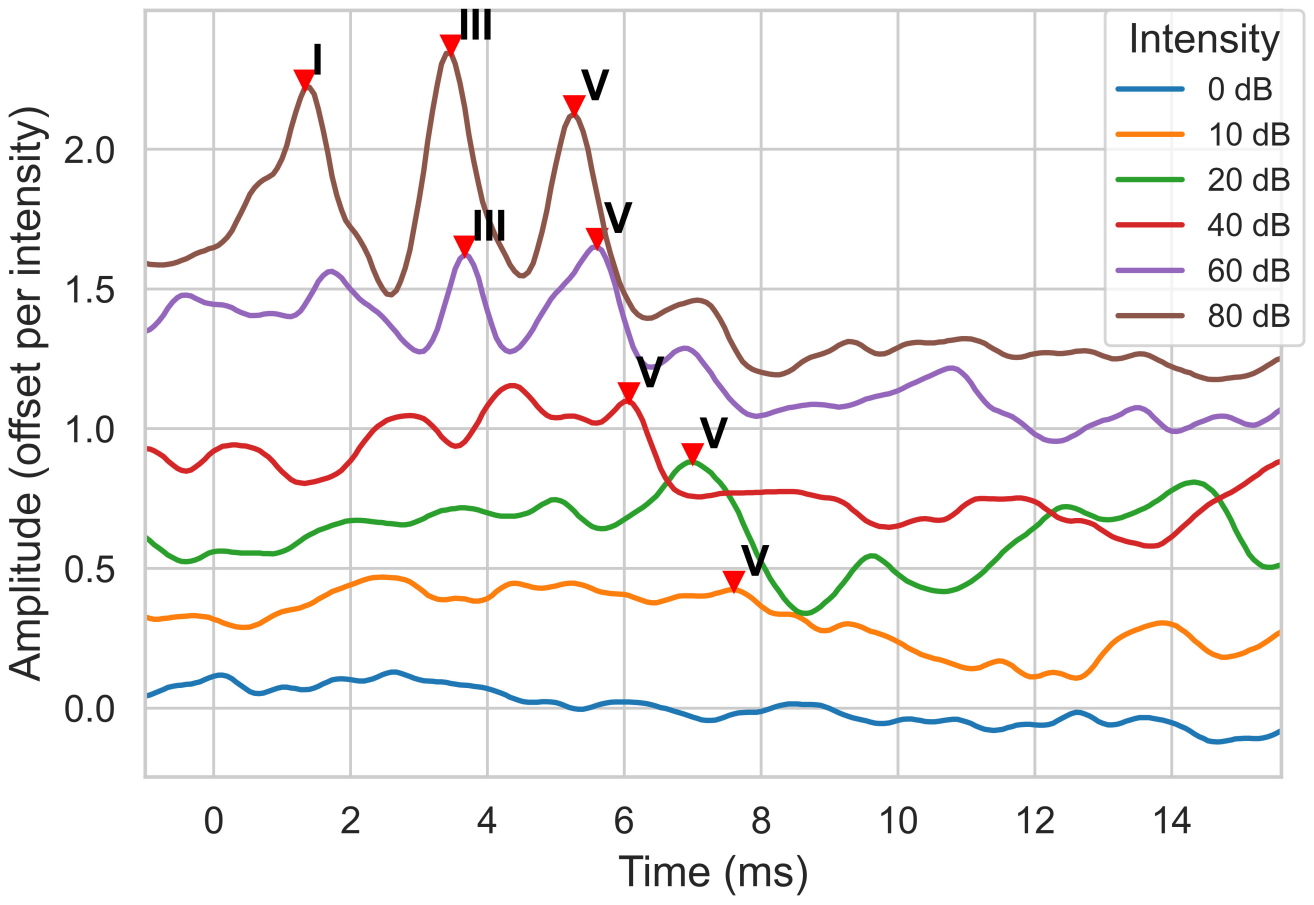
ABR signal examples for a normal hearing ear at different sound intensity (dB) levels. The signal amplitude is measured in μ*Volts*. Note that, to display all signals on the same plot, a signal is offset by 0.3 μ*V* from the previous one, starting with the 0 dB signal.

Auditory Brainstem Response (ABR) test is a widely used electrophysiological test method for detecting hearing loss, especially in infancy and individuals who cannot cooperate with behavioral tests (1). ABRs are recorded non-invasively via scalp electrodes and reflect the bioelectric activity of specific components of the auditory pathway following an auditory stimulus (2). Its clinical value lies in the objective assessment of hearing thresholds and functional integrity of the auditory system (3). An ABR signal has a typical structure with 7 positive waves. Three known waves (I, III, and V) are more prominent and therefore clinically the most useful (4). These waveforms are typically labeled through visual inspection. The lowest intensity level at which wave V can be observed gives the hearing threshold. An example is shown in Figure 1. The figure shows the ABR signals for six different intensity levels. The lowest intensity level where wave V is observed is 10 dB, indicating the hearing threshold of the ear.

Interpretation of an ABR test is performed by trained clinicians through visual inspection of ABR waveforms. Interpretation is known to vary greatly from clinician to clinician (5); and this subjectivity has encouraged the development of automated ways to interpret and analyze ABR signals. The initial work uses signal processing (6, 7) and traditional machine learning (8) techniques. In recent studies, deep learning based approaches have been explored. Some studies are designed to predict the latency (position) of the ABR waves. In (9), convolutional recurrent neural networks are used to localize waves I, III, and V using a dataset of 482 ABR waveforms recorded at 80 dB. In another study (10), an attention mechanism is used to predict the latency of wave V, achieving 95.89% accuracy and a maximum error of 0.1 ms (10).

Some other studies are designed to detect whether a wave (commonly, wave V) exists in an ABR signal. In (11), the so-called Wide&Deep and Light-MLP models, which use time-domain and frequency-domain features and incorporate demographic variables such as age, sex, and pure-tone thresholds, are used to detect the existence of wave V. The Wide&Deep model achieved a classification accuracy of 91.0%, while the Light-MLP model achieved 95.4%. Another study used a deep convolutional neural network to classify ABR waveforms into “clear response”, “inconclusive”, or “response absent”, achieving 92.9% accuracy along with high sensitivity and specificity on a public dataset (12). In (13), a convolutional neural network (CNN) based model is applied to standardized ABR waveform images to classify hearing loss, achieving 85% accuracy and contributing to earlier detection with improved diagnostic efficiency and objectivity (13). In (14), computer vision based approach is taken to detect Waves I, III, and V from ABR waveform images.

A summary and comparison of these recent deep learning approaches for automated ABR analysis is provided in Table 1. Unlike previous works focusing on wave V localization or existence classification, our approach jointly addresses both tasks using a unified deep learning framework. We propose a multi-task convolutional neural network that simultaneously classifies the presence of wave V and predicts its latency. This joint modeling helps the network learn richer representations and reduces overfitting, especially in low signal-to-noise scenarios.

**Table 1:** Comparison of recent deep learning studies for automated ABR analysis.

**Referernces**	**Model**	**Task**	**Input type**	**Performance**	**Sample size**
McKearney et al. (9)	CRNN	Waves I, III, V localization	1D waveform	*R*^2^ = 0.87	482 signals (80 dB, single center)
Ji et al. (10)	CNN + attention	Wave V latency prediction	1D waveform	96.76% accuracy; max error 0.1 ms	10,841 ABR data
Liang et al. (11)	MLP	Wave V presence classification	Waveform + demographics	92.5% accuracy (Light-MLP)	2,556 ABR data
McKearney and MacKinnon (12)	CNN	ABR response classification (clear / absent / inconclusive)	1D waveform	92.9% accuracy	232 paired ABR samples
Ma et al. (13)	CNN	Classify the presence or absence of hearing loss	Images	84.90% accuracy	10,000 samples
This study (2025)	Dual-input multi-task CNN (ResNet backbone)	Wave V classification + latency estimation (joint)	1D paired signals (test + 80 dB reference)	F1 = 0.92, *R*^2^ = 0.90	16,436 signals

Our method is further inspired by clinical practice, where audiologists often analyze ABR signals at multiple stimulus intensities, particularly higher intensities such as 80 dB, where wave V is more prominent. To emulate this reasoning process, our model receives as input a pair of ABR signals: the target signal and a reference signal from the same patient at a higher intensity level. This pairing strategy helps the model make better-informed decisions, particularly when wave V is difficult to detect at lower intensities.

To train and evaluate our models, we collected an in-house dataset comprising 16,436 auditory brainstem response (ABR) signals, of which 11,353 were recorded using the standard click stimulus. These recordings include data from both normal-hearing and hearing-impaired individuals. Each signal is paired with expert-annotated wave V latencies, providing high-quality labels for both classification (presence/absence) and regression (latency estimation) tasks. To the best of our knowledge, this is the first study to leverage multi-intensity signal pairs along with multi-task training for ABR analysis at this scale and level of precision.

In this paper, our contributions are as follows:

- We propose a novel approach that combines classification of wave V existence and localization into a single multi-task learning model.
- We introduce a signal-pairing technique, where each input signal is paired with a high-intensity (specifically, 80 dB) reference signal to provide contextual information inspired by clinical procedures.
- We employ a multi-task training approach that enables joint learning of both classification and latency prediction tasks.
- We investigate the effects of different loss functions on the training dynamics and the final model performance.

## 2 Methods

### 2.1 Data

**Figure 2:**
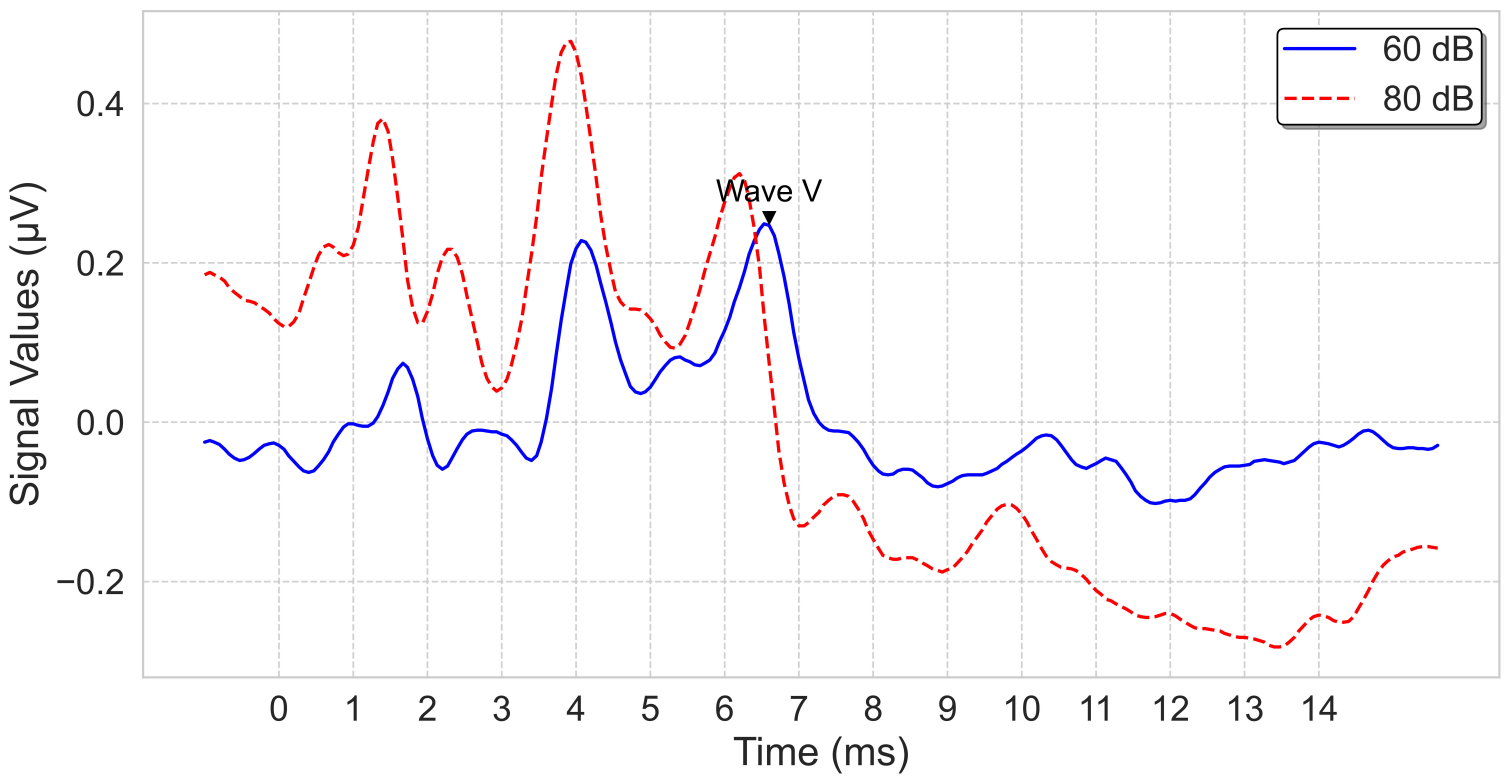
ABR signal examples, recorded at 60 dB and 80 dB.

The electrophysiological tests were performed in a sound-treated double-walled booth of Istanbul Medipol University—Mega Hospital with a bed and curtains to provide darkness to favor sleep. All data were collected with a commercial ABR software module (Interacoustics, software version 4.2.0.8) running on an Interacoustics Eclipse EP25 platform (hardware version 3.4.4). The click stimuli at alternating polarity, calibrated in dB normalized hearing level (nHL), were presented via insert phones. After cleaning the skin's surface with Nuprep gel, recording electrodes(Ag/AgCI) were placed on the forehead (vertex), the chin (ground) and the mastoids (i.e., reference electrode). Before starting to record, it was ensured that the impedance values of the electrodes were below 3–5 kOhms. Band-pass filtered from 100 Hz to 3,000 Hz using filter slopes of 12 dB/octave, and digitized with a 16-bit resolution. The sampling frequency is 15 kHz, which is about 0.067 milliseconds; the recording starts 1 ms before the click impulse and continues up to 20 ms after the impulse. An artifact rejection level of ±40 μ *V* was applied. The maximum intensity level was determined as 100 dBnHL, and waves I, III, and V were assessed at 80 dBnHL. Two runs, each consisting of averaged responses from 2,000 sweeps, were obtained at each presentation level, and thresholds were established using a 20-10 dB down and 5-10 dB up with steps that considered the last visible wave V as the threshold. Sample ABR recordings for an ear are shown in Figure 2.

Ethical approval for this study was obtained from the Ethics Committee for Non-Interventional Research of Istanbul Medipol University (Protocol number: E-10840098-772.02-785) on 26/01/2023.

### 2.2 Data Prepossessing

Our dataset comprises recordings from 934 patients (609 male, 325 female), including 1,717 ears. The type of hearing was classified into five groups: normal hearing, sensorineural hearing loss (SHL), profound hearing loss, conductive hearing loss (CHL), and neuropathy. Table 2 summarizes the overall distribution.

**Table 2:** Distribution of hearing types in the dataset.

**Hearing type**	**Right ear (count)**	**Left ear (count)**
Normal	400	404
SHL	184	180
Profound	170	178
CHL	87	84
Neuropathy	15	15
Total	856	861

In total, 16,436 ABR signals were recorded; about 31% of these waveforms lacked a discernible wave V, suggesting potential auditory pathway abnormalities. To prepare the data for the machine learning model, the first 250 samples were taken, which correspond to a time range from -1 ms to about 15.7 ms, beyond which the signal is irrelevant for wave V.

**Figure 3:**
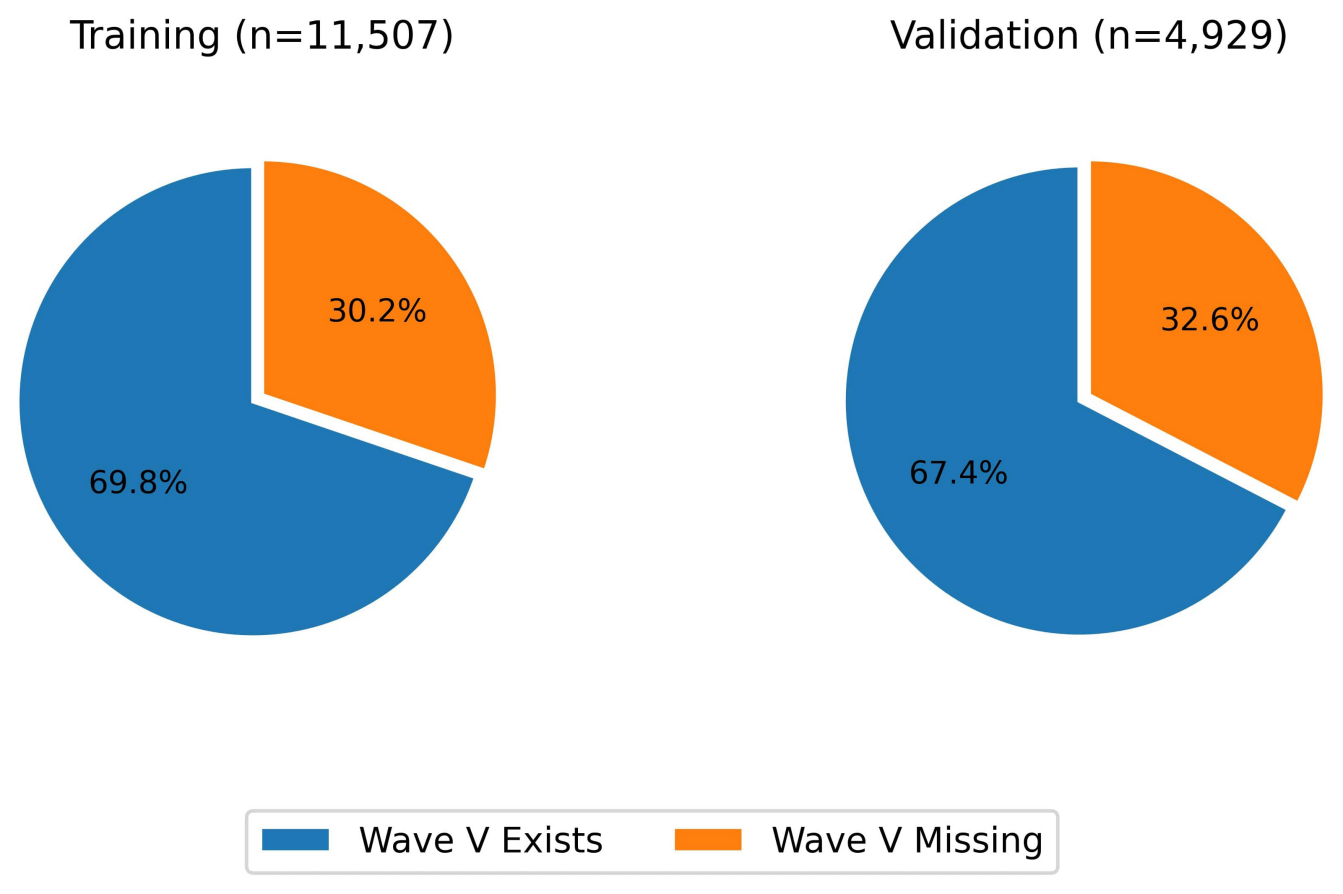
Distribution of wave V presence vs absence.

**Figure 4:**
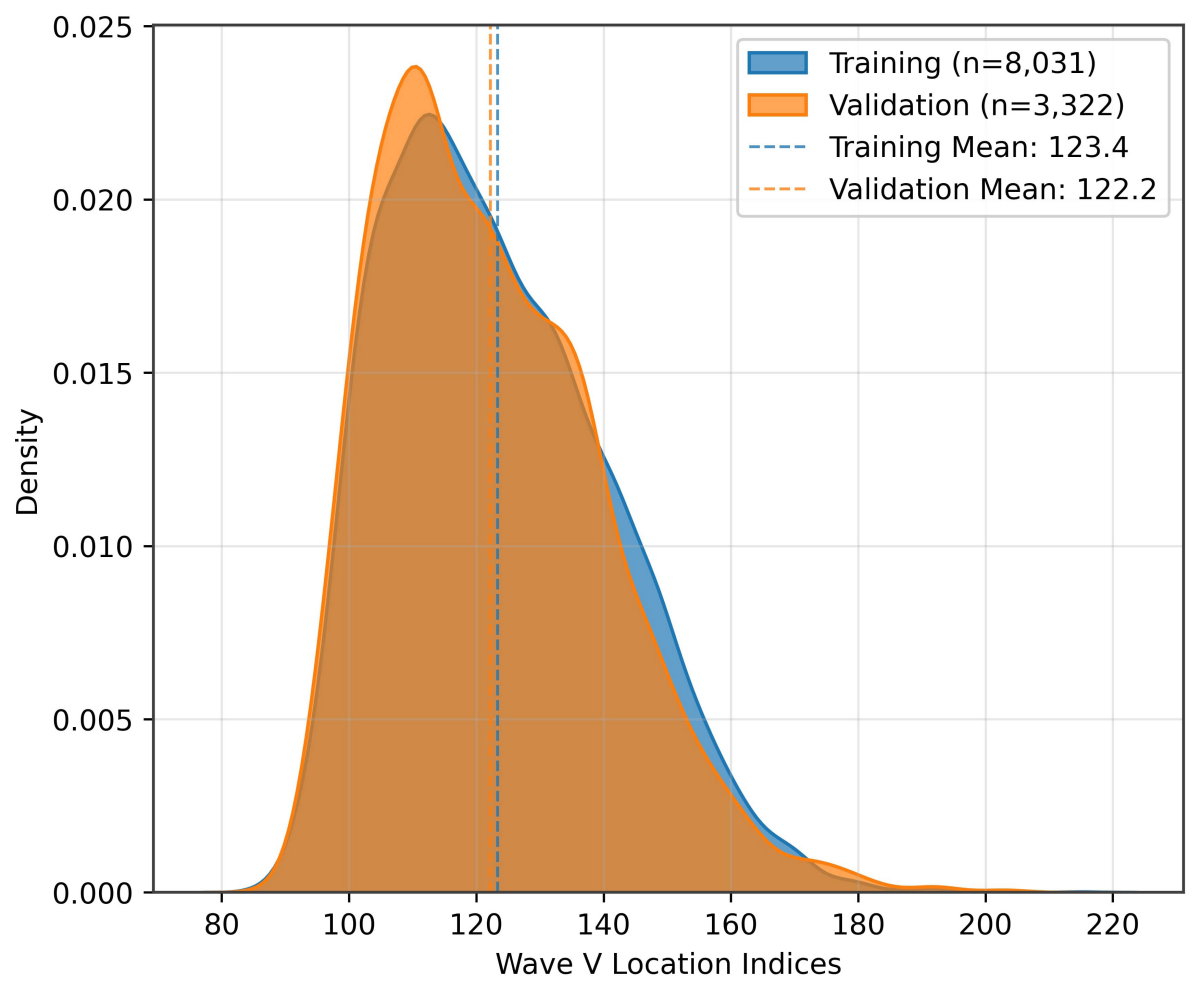
Distribution of wave V locations.

The dataset was then split into 70% training and 30% validation sets, preserving the ratio of wave V absence and presence. The split was done based on the ear; that means, it was ensured that signals from an ear are either in the training set or the testing set during the split. The final distributions with respect to the presence of wave V and with respect to the wave V positions are shown in Figures 3, 4, respectively.

### 2.3 Model architecture

**Figure 5:**
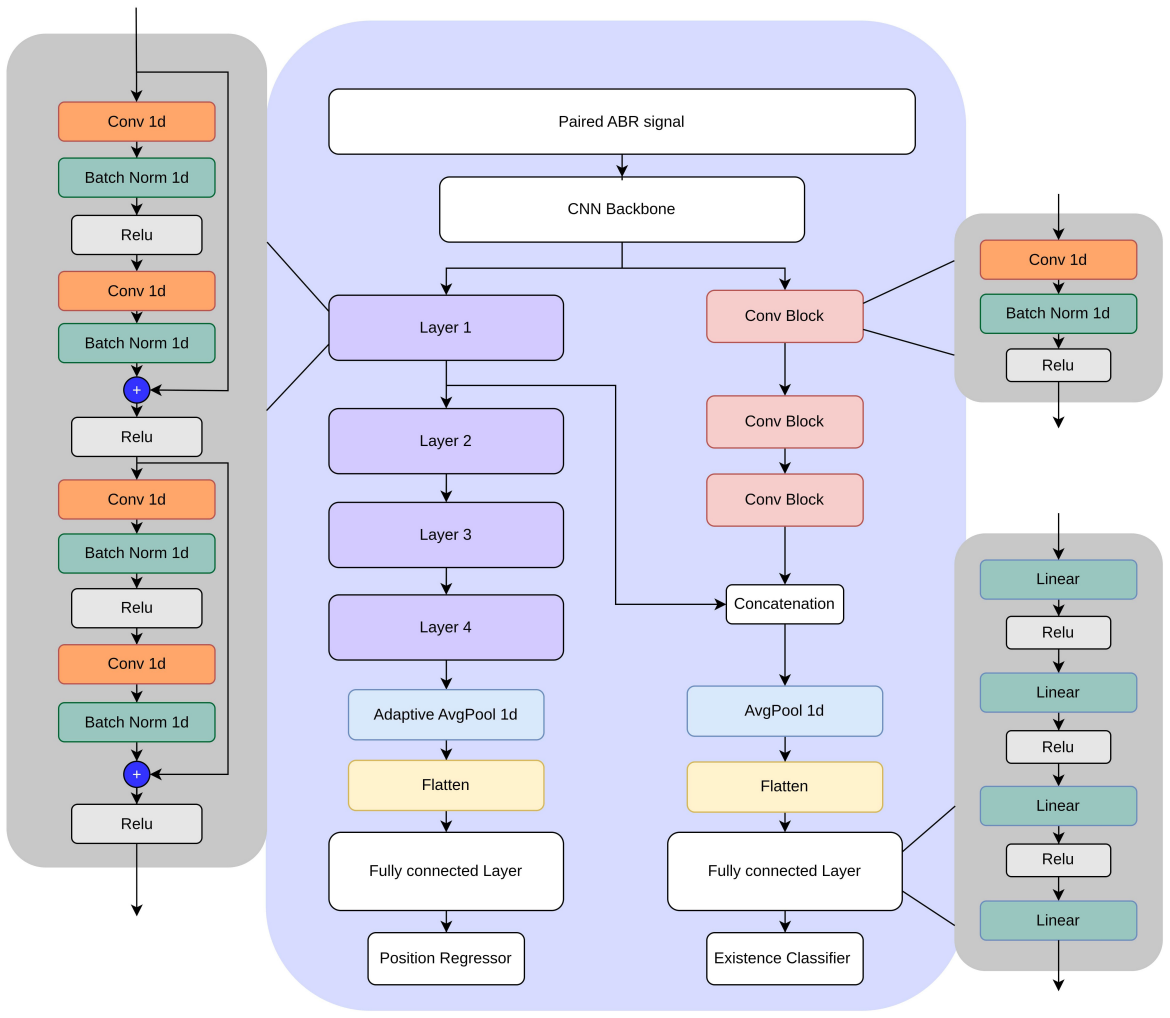
Multi-task model architecture.

In this study, we investigated convolutional neural networks (CNNs) (15) for simultaneous classification of wave V existence and regression of its latency in ABR signals. Our design process was iterative, starting with a simple 1D CNN and progressing toward a more advanced architecture that includes ResNet (16) blocks to improve performance and robustness. The final design is shown in Figure 5. The design includes a CNN backbone, followed by a regressor for wave V location and a classifier for wave V existence. The regressor head essentially has four ResNet-like layers. Each layer has a combination of convolution, batch normalization, and ReLU layers with skip connections. The standard residual blocks are modified to include temporal dilation rates of 2, 4, and 8, enabling multiscale feature extraction over varying latency spans. An adaptive average pooling layer is applied after the final residual block to aggregate temporal features before a fully connected layer.

The classification head uses a series of convolutional blocks, each combining convolution, batch normalization, and ReLU layers. To take advantage of the shared representations, intermediate feature maps from the regression head are concatenated to the convolution blocks before feeding them to the classification layer.

### 2.4 Paired signal approach

To enhance the model's ability to capture meaningful patterns, we introduced a paired-signal approach, in which an ABR recording (test signal) is paired with its corresponding high-intensity (specifically, 80 dB) reference signal from the same experiment. The paired signal is input to the model, which is designed to predict whether wave V exists in the test signal and its position. High-intensity ABR signals generally exhibit clearer waveforms, providing structural cues that facilitate the interpretation of lower-intensity responses. By pairing signals recorded from the same ear and session, we integrate complementary information that enhances wave V classification and latency prediction.

### 2.5 Loss functions for regression

To train the regression head, we investigated several loss functions. Two of those are the traditional mean squared error (MSE) and mean absolute error (MAE). For a batch size of *N*, the true value *y*_*i*_, and the predicted value ŷ_*i*_, the MSE loss and the MAE loss are


$${ℒ}_{\text{MSE}}=\frac{1}{N}\sum_{i=1}^{N}{\left({\hat{y}}_{i}-y_{i}\right)}^{2},$$ (1)

and


$$\begin{array}{l} L_{\text{MAE}}=\frac{1}{N}\overset{N}{\underset{i=1}{\sum }}|{\hat{y}}_{i}-y_{i}|, \end{array}$$ (2)

respectively.

In addition, we used a weighted squared error loss function, where deviation from the true value was penalized differently:


$$\begin{array}{l} L_{\text{W}}=\frac{1}{N}\overset{N}{\underset{i=1}{\sum }}w_{i}{\left({\hat{y}}_{i}-y_{i}\right)}^{2}. \end{array}$$ (3)

For the weights *w*_*i*_, we tested two functions. The first one is denoted as $L_{\text{W-sigmoid}}$, where the weights are based on a sigmoid function:


$$\begin{array}{l} w_{i}=1.0+\sigma \left(|{\hat{y}}_{i}-y_{i}|-m\right). \end{array}$$ (4)

**Figure 6:**
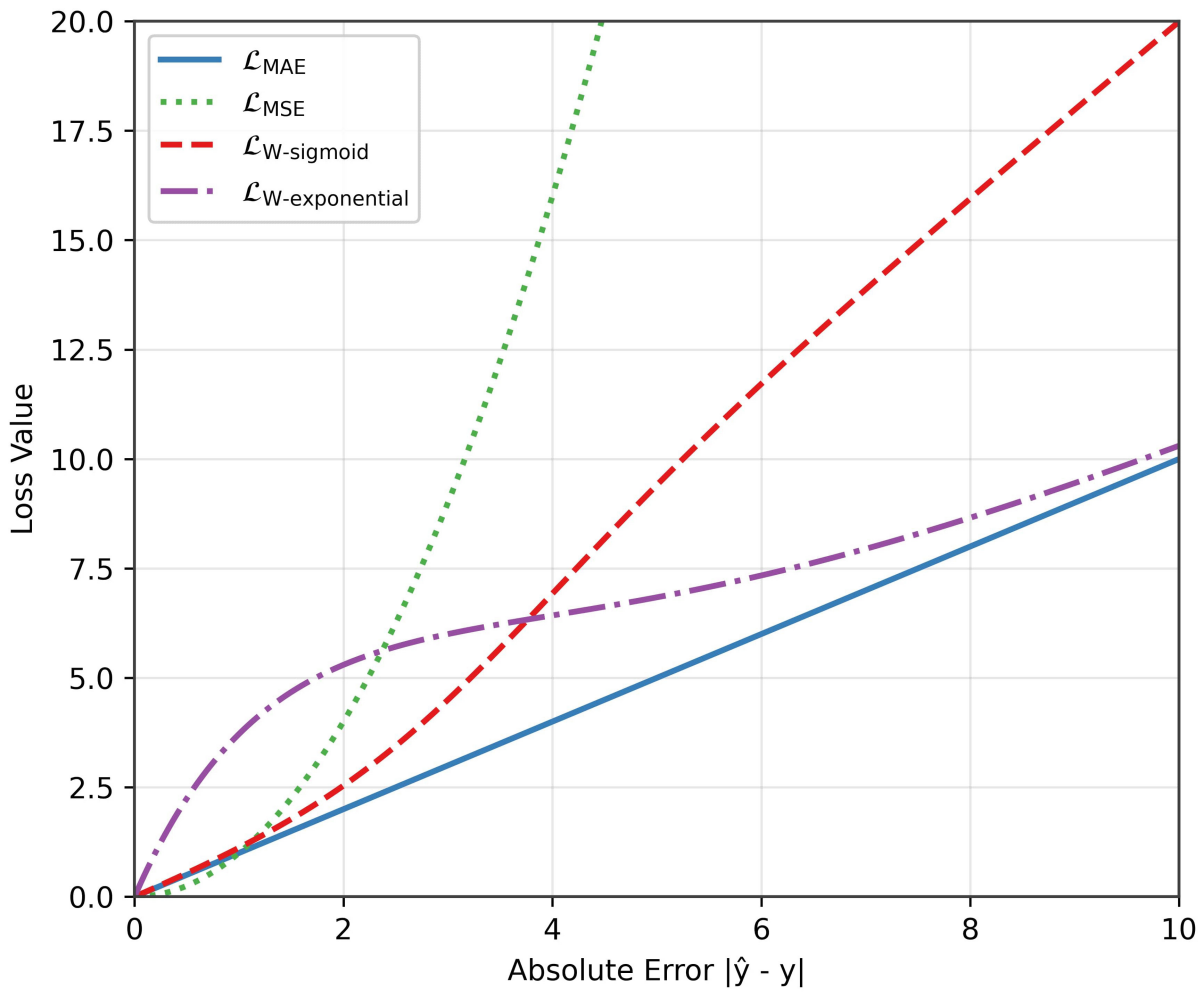
Comparison between different loss functions for the regression task.

The second one is denoted as $L_{\text{W-exponential}}$, where the weights are based on an exponential function:


$$\begin{array}{l} w_{i}=1.0+\exp\left(-k\cdot \left(|{\hat{y}}_{i}-y_{i}|-m\right)\right), \end{array}$$ (5)

with *m* and *k* being some scalar values. The behaviors of these functions ($L_{\text{MSE}}$, $L_{\text{MAE}}$, $L_{\text{W-sigmoid}}$, $L_{\text{W-exponential}}$) are plotted in Figure 6 for *m* = 3 and *k* = 0.5. In the case of $L_{\text{W-sigmoid}}$, the function behaves similarly to the MAE loss for small deviations, but for large deviations, it penalizes more than the MAE loss. The exponential loss has the opposite behavior: It penalizes more than the MAE loss when the deviation is small, but behaves like the MAE loss as the deviation gets larger.

### 2.6 Loss functions for classification

To train the classification head to detect the presence or absence of wave V in an ABR signal, we employed the binary cross-entropy (BCE) loss, a standard choice for binary classification tasks. For each sample *x*_*i*_ with ground-truth label *y*_*i*_∈{0, 1} (where 1 denotes the presence of wave V and 0 denotes the absence) and predicted probability ŷ_*i*_, the BCE loss is defined as:


$$\begin{array}{l} L_{\text{BCE}}=-\frac{1}{N}\overset{N}{\underset{i=1}{\sum }}\left[y_{i}\log\left({\hat{y}}_{i}\right)+\left(1-y_{i}\right)\log\left(1-{\hat{y}}_{i}\right)\right], \end{array}$$ (6)

which essentially measures how well the predicted probabilities match the true labels.

### 2.7 Training process

To maximize clinical utility, we employed a multitask learning framework (17) that simultaneously predicts wave V latency (regression) and classifies its presence (binary classification). Our training procedure consisted of three stages. First, we trained the latency prediction branch to convergence. Next, we froze its convolutional backbone and shared layers, and used these pre-trained features to initialize the classification branch (the right side of Figure 5). Finally, we unfroze all network parameters and fine-tuned the entire model using a combined loss function, which is a weighted sum of classification loss and regression loss functions. In the experiments, the weight of the classification loss is set to double the weight of the regression loss. This multi-task approach allows the model to learn shared representations that benefit both tasks, enhancing the overall performance and robustness of the system.

### 2.8 Hardware configuration

Inference speed was evaluated on the system described in Table 3. On the GPU, the model achieves an average inference time of 3.31 ms per paired ABR signal (median: 3.14 ms, std: 0.58 ms), corresponding to a throughput of 303 samples/second. On CPU, inference takes 5.37 ms on average (median: 5.29 ms, std: 0.55 ms), corresponding to 186 samples/second. These results confirm that the model is suitable for near real-time clinical use.

**Table 3:** Hardware specifications used for training and testing.

**Component**	**Specification**
Processor	13th Gen Intel^®^ Core™ i7-13700H (2.40 GHz)
RAM	16.0 GB
GPU	NVIDIA GeForce RTX 4050 Mobile (6 GB VRAM)
OS	Ubuntu 22.04 LTS (64-bit)

## 3 Results

The performance of the proposed model is evaluated on the test set using several metrics. To show the effectiveness of the multi-task approach, we also evaluated single-task CNN and single-task ResNet approaches.

The single-task CNN consists of CNN layers followed by a fully connected layer that ends with a regressor for wave V latency prediction or a binary classifier for classifying the existence of wave V. The model is separately trained for the regression and regression tasks, and compared with the proposed multi-task approach.

The single-task ResNet model is essentially the regressor side of the multi-task model shown in Figure 5. It consists of a CNN backbone followed by multiple layers of residual blocks. It is trained for the regression task and compared with proposed multi-task approach.

For all models, we investigated the effect of having a paired input instead of a single input. The input layer is modified to take a paired or single input, keeping the rest of the network unchanged. Each model is separately trained for the same number of epochs to ensure a fair comparison.

The models with trained weights are available at https://github.com/youssefdarahem/ABR_analysis_model.

### 3.1 Performance metrics

To measure regression performance, we used three performance metrics. The first two are the mean absolute error (MAE) and the coefficient of determination (*R*^2^), which are commonly used metrics in regression problems. The third metric is the “accuracy for a given error tolerance”, which is the percentage of predictions within a specified error margin (tolerance) of the ground truth value. As tolerance increases, accuracy improves due to a wider margin of error. High accuracy at low tolerances indicates superior model performance.

To measure classification performance, we use standard classification metrics, including accuracy, precision, recall, F1 score, and area under the curve (AUC).

### 3.2 Results and comparisons

The regression results are shown in Table 4 (The regression loss function used for the results in this table is the weighted squared error with exponential decay). Three different models are evaluated. For each model, single-input and paired-input versions are trained and tested. For each model, it is seen that the paired-input version produces better *R*^2^ and MAE results compared to the single-input version, supporting the hypothesis that the use of multiple ABR signals helps to distinguish the locations of the waves. Regarding the accuracy for different tolerances, it is observed that the paired-input versions provide more gains for smaller tolerances.

**Table 4:** Regression (prediction of wave V latency) results.

**Model**	**R^2^**	**MAE**	**Accuracy for different tolerances (ms)**				
			**0.06**	**0.12**	**0.18**	**0.24**	**0.60**
Single-task CNN regressor (single input)	0.53	8.77	0.09	0.17	0.26	0.33	0.66
Single-task CNN regressor (paired input)	0.56	8.23	0.09	0.18	0.27	0.35	0.71
Single-task ResNet regressor (single input)	0.85	2.27	0.67	0.82	0.87	0.90	0.95
Single-task ResNet regressor (paired input)	0.90	1.94	0.70	0.83	0.88	0.91	**0.96**
Proposed multi-task model (single input)	0.85	2.05	0.75	0.86	0.89	0.91	0.95
Proposed multi-task model (paired input)	**0.90**	**1.72**	**0.81**	**0.88**	**0.90**	**0.92**	0.95

Bold values indicate the best (highest or lowest, as appropriate) performance among all methods compared in each row.

**Figure 7:**
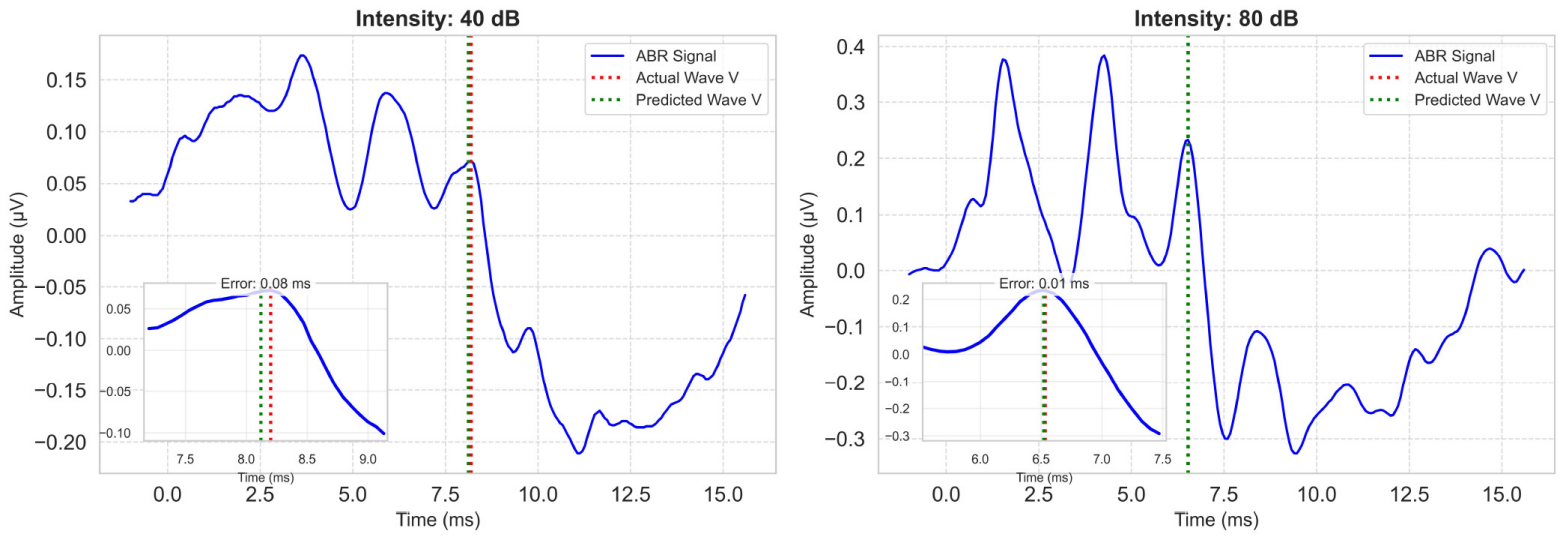
Example model predictions for a low and a high intensity ABR signal. Each plot includes the zoomed-in region of Wave V. Ground-truth Wave V latency is marked with a red vertical line; model prediction is shown in green.

Considering all metrics, the best performing model is the proposed multi-task model, with an *R*^2^ value of 0.90, MAE value of 1.72 index error, which is about 0.11 ms. Representative model outputs are visualized in Figure 7, demonstrating accurate Wave V localization even in low-intensity or noisy recordings, thanks to the paired-input context.

The classification performances are summarized in Table 5. The proposed model outperformed the single-task CNN model in all metrics, including accuracy, precision, recall, and F1 score, demonstrating the benefits of deeper feature extraction and regularization. These results underscore the value of advanced neural architectures in improving ABR signal classification. For both models, the paired-input version produces better results compared to the single-input version.

**Table 5:** Classification (prediction of wave V existence) results.

**Model**	**Accuracy**	**AUC**	**F1-score**	**Recall**	**Precision**
Single-task CNN classifier (single input)	0.82	0.90	0.87	0.87	0.87
Single-task CNN classifier (paired input)	0.84	0.90	0.88	0.86	0.90
Proposed multi-task model (single input)	0.87	0.94	0.90	0.89	0.91
Proposed multitask model (paired input)	**0.89**	**0.96**	**0.92**	**0.91**	**0.93**

Bold values indicate the best (highest or lowest, as appropriate) performance among all methods compared in each row.

## 4 Analysis

We have done analyses on the effects of loss functions, architectural choices, and click intensities.

### 4.1 Effect of loss function

**Figure 8:**
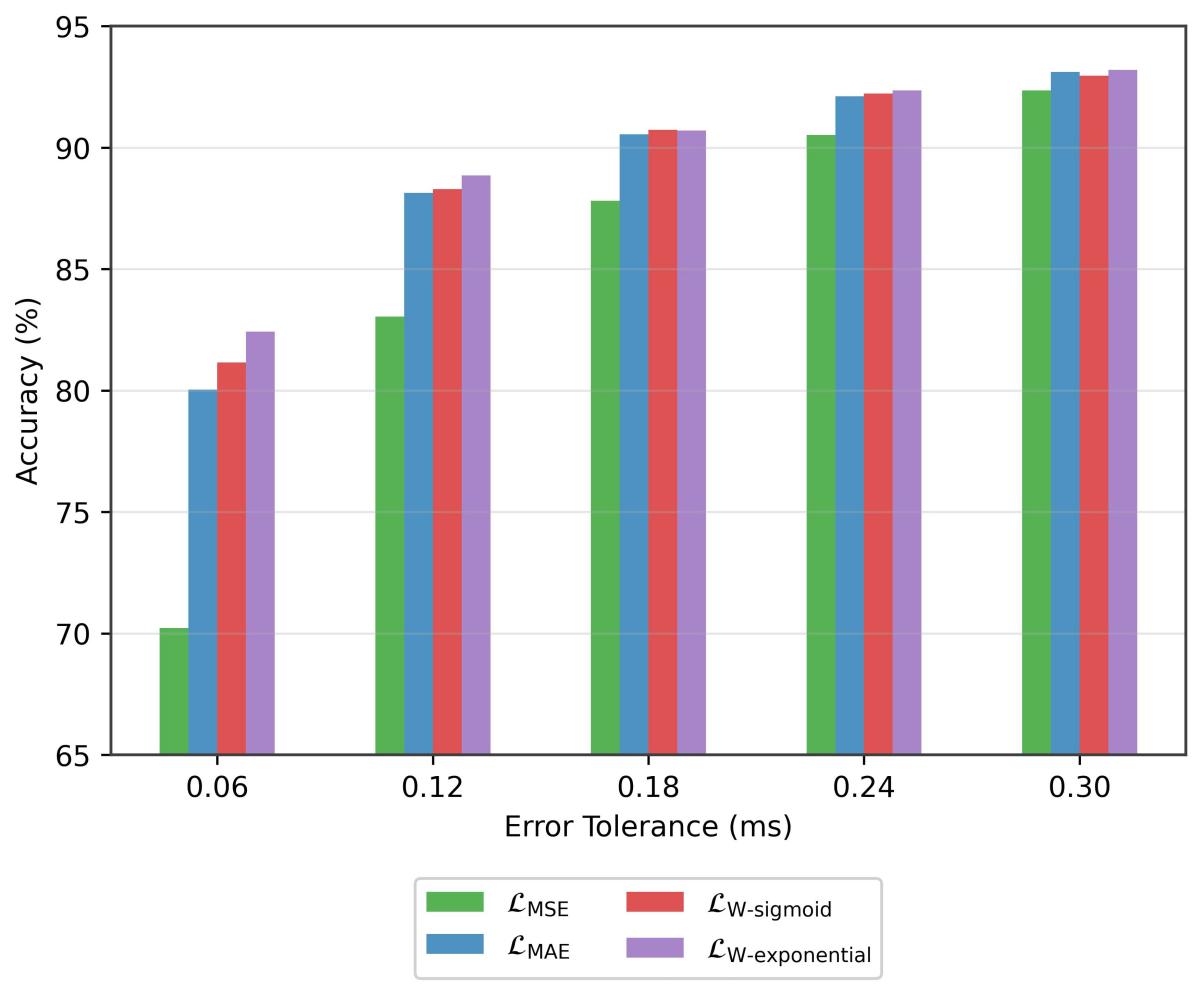
Accuracy achieved with different loss functions.

On the regression head of the model, we investigated the use of alternative loss functions. The accuracy results for different tolerance values are shown in Figure 8. In all cases, the MSE loss leads to the worst performance. The MAE loss provides marked improvement over the MSE loss, especially in the low error tolerance scenarios (i.e., 0.06 ms and 0.12 ms). The best performance overall is achieved with the weighted loss that uses an exponential function and behaves somewhere between MSE and MAE loss as shown in Figure 6.

### 4.2 Effect of intermediate-level feature sharing

A regular multi-task network has a common backbone followed by multiple separate heads. In this work, we investigated a variation where there is sharing in the intermediate layers. Specifically, as shown in Figure 5, an intermediate tensor from the regression head is fed to the classifier head, transferring important features. This, in effect, reduces the need for a more complex classifier head and leads to better performance with limited data. We tested connections from different intermediate levels and achieved the best performance when low-level features were transferred. The network is trained with and without the connection, and the results are shown in Table 6.

**Table 6:** Effect of the intermediate connection in the proposed model.

**Model**	**Accuracy**	**AUC**	**F1-score**	**Recall**	**Precision**
**Without connection**	**0.88**	**0.95**	**0.90**	**0.88**	**0.94**
With connection	**0.89**	**0.96**	**0.92**	**0.91**	0.93

Bold values indicate the best (highest or lowest, as appropriate) performance among all methods compared in each row.

### 4.3 Effect of stimulus volume

**Figure 9:**
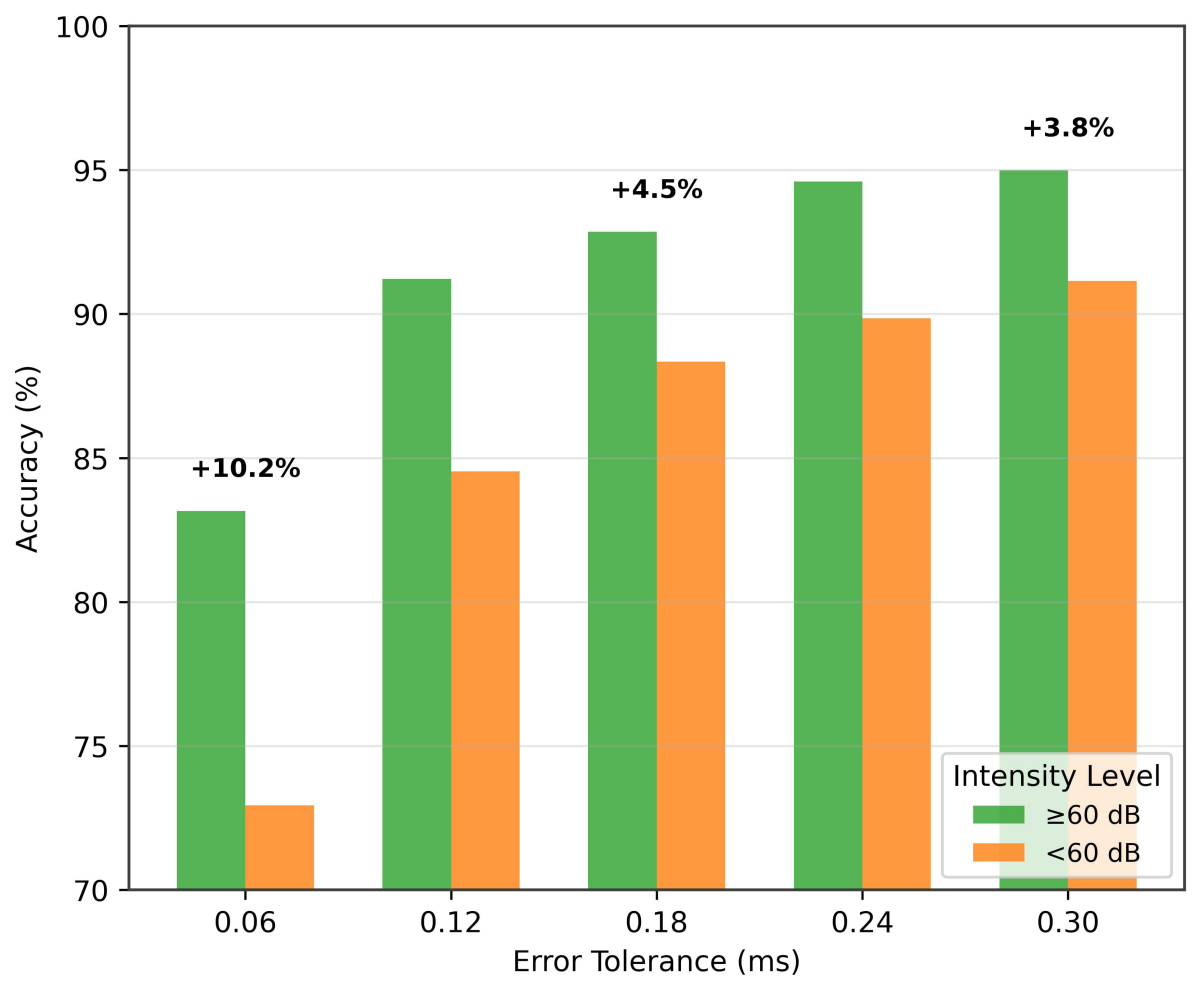
Wave V localization performance for different intensity (dB) levels.

As the stimulus volume decreases, wave V becomes more difficult to detect in the ABR signal. In the previous section, we reported results without making any distinction of the stimulus intensity. Here, we analyze the performance of low-intensity and high-intensity groups. We divided the test data into two groups, namely, high intensity (≥60 dB) and low intensity (< 60 dB) groups. Figure 9 shows the wave V localization results; as expected, the accuracy is significantly higher for the high-intensity group. A similar trend is also observed for the classification performance, as shown in Table 7.

**Table 7:** Classification performance by intensity (dB) level.

**Intensity**	**Accuracy**	**Precision**	**Recall**	**F1 score**
** < 60dB**	**0.87**	**0.95**	**0.88**	**0.91**
≥60dB	**0.92**	**0.95**	**0.91**	**0.93**

Bold values indicate the best (highest or lowest, as appropriate) performance among all methods compared in each row.

These results also support the idea of a paired input approach. A high-intensity reference signal, which has prominent wave characteristics, provides important information for localizing and classifying the low-intensity signal.

## 5 Conclusions

In this study, we presented a multi-task deep learning model to address two critical challenges in ABR analysis: The detection of wave V latency and classification of its presence. By unifying classification and regression into a single architecture and optimizing both tasks together, we achieve greater robustness and precision than separate, single-task models. We presented a paired-input approach, and investigated various loss functions for training.

Key findings include the following.

- **Paired-input approach**: The paired-input strategy provides the network with complementary information: the high-intensity waveform acts as a clear reference in which Wave V is readily visible, guiding the interpretation of noisier, low-intensity recordings. Clinically, audiologists routinely compare high-intensity and low-intensity responses when identifying wave V; our method emulates this practice and embeds it directly into the model.

Incorporating high-dB reference signals alongside target ABR signals enhanced the model's ability to detect subtle patterns, improving localization accuracy by 6% and classification performance, particularly in low-intensity (≤60 dB) scenarios.

- **Loss functions**: To encourage high precision around clinically relevant latency thresholds, we evaluated two new loss functions in addition to the MAE and MSE losses. The new loss functions place relatively more weight on either small errors or large errors during training.

The loss function, which is tailored to focus on tight error margins, yields more reliable latency estimates for diagnostic purposes. The model achieves around 88% accuracy for an error margin of 0.12 ms, and 81% accuracy for an error margin of 0.06 ms.

- **Multi-task model with common backbone and shared layers**: Our multi-task architecture leverages shared features for presence detection and latency regression. After pretraining the regression branch, we fine-tuned the full network with a combined loss, allowing the classifier and regressor to reinforce each other's learned representations. This synergy yields notable gains, especially in localization performance, while aligning closely with clinical workflows by delivering both outputs in a single inference pass.

The model achieved an F1 score of 0.92 for classification and an *R*^2^ of 0.90 for latency prediction, surpassing single-task models.

Automated analysis of ABR signals offers an objective and efficient way of diagnosing auditory disorders. Future work may explore broader datasets, real-time implementation, and integration with clinical devices. This work underscores the potential of deep learning in advancing electrophysiological diagnostics, bridging the gap between computational models and practical audiological care.

## Data Availability

The original contributions presented in the study are included in the article/supplementary material, further inquiries can be directed to the corresponding author.
